# Experimental evaluation of bamboo fiber/particulate coconut shell hybrid PVC composite

**DOI:** 10.1038/s41598-021-85038-3

**Published:** 2021-03-09

**Authors:** Adeolu A. Adediran, Abayomi A. Akinwande, Oluwatosin A. Balogun, O. S. Olasoju, Olanrewaju S. Adesina

**Affiliations:** 1 Department of Mechanical Engineering, Landmark University, PMB, Omu-Aran, 1001 Kwara State Nigeria; 2 Department of Metallurgical and Materials Engineering, Federal University of Technology, Akure, Ondo State Nigeria; 3 Faculty of Material Science and Chemical Engineering, Ningbo University, Ningbo, China

**Keywords:** Materials science, Structural materials, Engineering, Civil engineering

## Abstract

Bamboo fibers (BF) treated in 1.3 Molar NaOH and particulate coconut shell (PCS) sieved to − 45 µm were incorporated into polyvinyl chloride (PVC) matrix towards improving the properties of PVC composite for ceiling boards and insulating pipes which sags and degrade with time needing improvement in properties. The process was carried out via compression moulding applying 0.2 kPa pressure and carried out at a temperature of 170 °C. Composites developed were grouped according to their composition. Groups A, B, C, and D were infused with 2, 4, 6 and 8 wt% PCS at constant amount, respectively. Each group was intermixed with a varying proportions of BF (0–30 wt% at 5% interval). Tests carried out on the samples produced revealed that the yield strength, modulus of elasticity, flexural strength, modulus of rupture were enhanced with increasing BF proportion from 0 to 30 wt% BF at 2 wt% constant PCS input. Thermal and electrical properties trended downward as the fiber content reduced even as the hardness was enhanced with PCS/BF intermix which was also reflected in the wear loss index. Impact strength was highest on the infix of 4 wt% PCS and 15 wt% BF. Compressive strength was better boasted with increasing fiber and PCS amount but 8 wt% PCS amounted to depreciation in trend. It was generally observed that PCS performed optimally at 2 wt% incorporation while beyond that resulted in lowering of strength. Blending of the two variable inputs; 0–30 wt% BF and 2 wt% PCS presented better enhancement in properties.

## Introduction

In recent years, composites are designed to achieve certain configurations and requirements (for structural, electronic packaging, automobile, aerospace, and household applications). These composites possess improved strength to weight properties, low density, and they are relatively cheap. Owing to increase in the amount of composite materials being adopted globally, researches are being tailored towards the development of biodegradable filler reinforced composites. Bio-fillers possess improved specific strength and modulus and impact as a measure of resistance to crack propagation when used as reinforcements in a polymer matrix. In addition, the overall cost of composite production is reduced^1–3^.

Composites materials are engineered by the combination of two or more distinct constituents, where one of the constituents is referred to as the matrix material and the other is known as the reinforcing material. Additionally, embedding of the reinforcing phase into the matrix material is done at a macroscopic scale. The distinct phases maintain their inherent physical and chemical properties. Matrix material serves the role of shielding the fibers from chemical and environmental attacks (like sunlight, heat, and moisture). They keep the fibers in place while ensuring effective load transfer among fiber strands. The toughness of the composite formed depends on the type of matrix adopted^4,5^. In addition, the matrix gives aesthetic value and good surface finish to the developed composite. On the other hand, the reinforcing phases could serve as the major load carrying component^6^.

Numerous studies have highlighted the importance of various bio-fillers (majorly from agro-products) and their environmental advantages which include reduced consumption of nonrenewable materials and lowering of greenhouse gas emissions which in turn reduce environmental pollution^7–10^. Natural fibers like bamboo^11^, jute^12^, oil palm^13^, cotton^14^, and sisal^15^ amongst others are ligno-cellulose based materials which are environmentally friendly substitutes over the synthetic (Kevlar, glass and carbon) fibers being used in time past^16,17^. Other attractive properties of natural fibers are good toughness, renewability, biodegradability, cost effectiveness, good specific strength, light weight, availability, non-toxic in nature, and the tools used in processing them are not abraded^18,19^.

Howbeit, natural fillers possess several disadvantages such as poor adhesion with the polymeric matrix owing to their hydrophilic nature, they possess the tendency to degrade when employed in high temperature applications^20,21^. These fillers are usually of inhomogeneous dimensions, they have low melting point and high moisture absorption characteristics which culminate in degradation while in use. Literatures have singled out the importance of chemical treatments in reducing the hydrophilicity of natural fillers and improving the interfacial adhesion created between the hydrophobic matrix and reinforcement^22,23^. Examples of chemical treatments used in overcoming the aforementioned disadvantages are alkalization^24,25^, benzoylation^26^, acrylation^27^, silane^28^, and flame retardant treatments^29,30^.

The use of bamboo is attracting significant attention by dint of their availability, good mechanical characteristics, recyclability, and their service performance can be compared to that of synthetic fiber. Bamboo wastes have been used in the form of fibers, particulates, and ash. A study carried out by Ref.^31^ showed that chemical treatment of 0.5 M NaOH solution effectively modified the surface of bamboo fiber by increasing the number of sites available for mechanical interlocking with high density polyethylene matrix. This culminates in improved mechanical properties. Optimum performance was achieved at 2–4 wt% bamboo fiber addition. The research of^32^ studied the effect of alkali treatment on bamboo fibers at 2, 6, and 10% NaOH solution for a period of 12 h. The study highlighted that 6% NaOH showed the optimum performance for the development of bamboo fiber reinforced composite, which was also adopted in this study. The study also established the fact that mechanical properties such as fracture toughness and flexural modulus depend on fiber length. Where increase in the length of bamboo fibers was accompanied by a corresponding increase in the aforementioned properties. Thermal stability and biodegradability of matrix can be improved with the addition of bamboo fiber as reported by Ref.^33^ who reported improved flexural strength, water absorption characteristics. Decreased in weight loss was observed when the samples were buried in the soil. Furthermore, the addition of bamboo fiber to polypropylene showed improvement in mechanical properties up to 50% by weight of the matrix as revealed^34^. Considerable efforts toward improving the performance of bamboo show that the use of compactibilizers such as maleated elastomer modifier in composites shows better properties compared to unmodified samples^35,36^.

Contemporary studies have shown that fiber and particulate reinforcement can be combined to improve the properties of polymeric matrix by the formation of hybrid composites with precise configuration, which has been a major focus of research in the last 20 years^37–39^. Nevertheless, none had considered intermix of bamboo fiber and particulate coconut shell in PVC matrix; a feat implemented in this study towards property enhancement of PVC.

Coconut shell is a non-edible hard part of coconut, which is widely regarded as waste and dumped in landfills. This shell possesses good strength and modulus, it is rigid and usually grown in the tropical areas of Africa. Coconut shell in previous researches has shown the ability to improve the compressive strength when incorporated into epoxy matrix^40^. More so^41^, proved that treatment of coconut shell with 1% NaOH solution increased the mechanical properties and thermal stability of unsaturated polyester when homogenous dispersion was achieved between the matrix and reinforcement. PVC is light, with good electrical insulation, corrosion and weathering resistance, abrasion resistance and cost effective. As a result of the good electrical insulation property its used as insulation pipes in running electrical pipes, also owing to good thermal insulation, it is employed in celing board application. However, these pipes are observed to degrade with time; evident in sagging and cracking before eventual failure of this pipes. This is ascribable to low mechanical properties which can be improved by the incorporation of fiber and particulate as noted in previoud study^42–44^. Therefore, from the literature review and the salient properties exhibited by bamboo fiber and coconut shell particulate, this study considered it a worthwhile to study the influence of these reinforcements at varied weight fraction on the properties of polyvinyl chloride towards improving properties of PVC base material for ceiling board and insulation pipes material.

## Materials and methods

### Materials and processing

Materials utilized in this study include sodium hydroxide, hydrochloric acid, bamboo fiber, coconut shell, and polyvinyl chloride pellets. Prior to chemical treatment, bamboo fibers (properties highlighted in Tables 1 and 2) and shells obtained as wastes were sundried for 7 days, treated, and used for composite development. Similar to procedure employed in^45,46^, bamboo fiber was treated with 1.3 Molar NaOH solution, the medium for a period of 12 h and then washed with distilled water followed by sun drying for 7 days. Similar to^45^, coconut shell was treated with of 1.3 Molar sodium hydroxide for impurities removal after which the shell was washed in water at 50 °C and sundried for 3 days to ensure complete dryness. Sequel to this was the grinding, pulverizing and sieving of the shell using using laboratory sieve shaker in line with ASTM D6913-17^47^ to obtain a coconut shell particle size of − 45 µm which was used along with bamboo fiber as reinforcing materials in polyvinyl chloride matrix.

**Table 1:** Properties of bamboo fibers used.

Parameter	Length	Diameter	Aspect ratio
Value	20 mm	0.25 mm	80

**Table 2:** Chemical composition of treated bamboo fiber.

Composition	Amount before NaOH treatment (%)	Amount after NaOH treatment (%)
Cellulose	52.3	58.97
Hemicellulose	23.7	19.5
Lignin	14.6	10.3
Ashes and water content	7.4	4.6
Other	2.0	6.63

The parameter (length, diameter and aspect ratio) for the bamboo fiber used in the course of the study is highlighted in Table 1. Table 2 higlights the chemical composition of bamboo fiber before and after treatment. Results content having highest proportion revelaed increase in cellulose content, reduction in hemicellulose, lignin and ash and water content.

**Figure 1:**
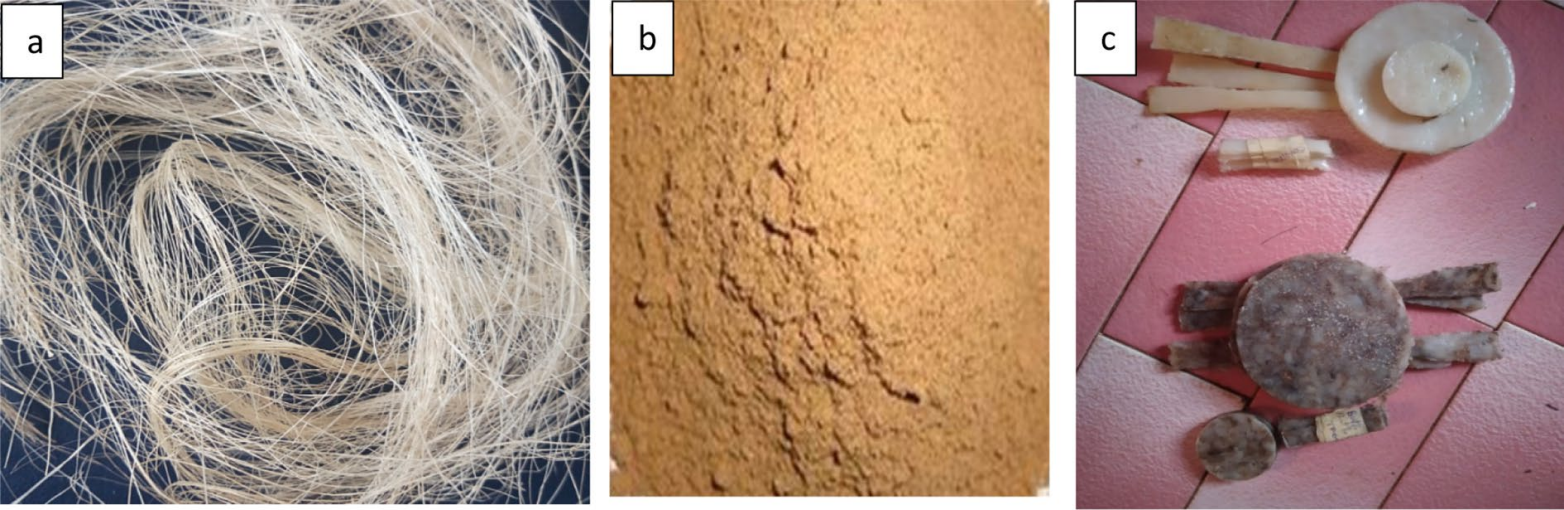
Pictures of (**a**) bamboo fiber (**b**) coconut shell powder **c** some composites samples developed.

Table 3 reveals the chemical composition of coconut shell powder with silica (SiO_2_) sharing the highest content followed by alumina (Al_2_O_3_) which are strength enhacing media, contributiong to strength of composite. Figure 1a–c show the pictorial representative of the bamboo fiber, coconut shell powder and the composites developed respectively.

**Table 3:** Properties of coconut shell powder.

Compound present	Coconut shell powder
SiO_2_	53.67
CaO	5.56
Al_2_O_3_	11.12
Fe_2_O_3_	6.21
Na_2_O	1.14
MnO	1.35
P_2_O_5_	0.22
MgO	3.08
Na_2_O	1.14
Others	8.92
LOI	7.59

*LOI* loss on ignition.

### Composite development

Cast iron moulds with dimensions of 150 × 50 × 3 (mm^3^) and dumb bell shape, mould cavity of 3 mm thickness and length of 150 mm were adopted for the production of specimens used for the evaluation of flexural and tensile properties respectively. Petroleum jelly was applied on the mould surfaces for easy removal of specimens. Prior to composite production, the moulds used were cleaned to remove the residual polymeric material present on the surface of the mould.

Bamboo fiber and particulate coconut shell were incorporated into polyvinyl chloride matrix in varying proportions as categorized in group A (containing 2 weight percent (wt%) proportion of CSP and varying proportion of BF at 0–30 wt%), group B (containing 4 wt% proportion of CSP and varying proportion of BF at 0–30 wt%), group C (containing 6 wt% proportion of CSP and varying proportion of BF at 0–30 wt%) and group D (containing 8 wt% proportion of CSP and varying proportion of BF at 0–30 wt%) as represented in Table 4. Compression moulding machine was used to produce pure polyvinyl chloride and hybrid BF/CSP composites. Specimens were compressed at 170 °C for a period of 10 min while employing a pressure of 0.2 kPa. Teflon sheet was used to cover the upper and lower part of the mould to avoid burning of the composites which may result from direct heating of metallic plates while petroleum jelly was applied on the mould surface for easy removal of specimens.

**Table 4:** Mix proportion.

Group A (2 wt% CSP)			Group B (4 wt% CSP)			Group C (6 wt% CSP)			Group D (8 wt% CSP)		
BF	CSP	PVC	BF	CSP	PVC	BF	CSP	PVC	BF	CSP	PVC
**Mix proportion of specimen produced at varying content of BF and CSP**											
0	3	97	0	6	94	0	9	91	0	12	88
5	3	92	5	6	89	5	9	86	5	12	83
10	3	87	10	6	84	10	9	81	10	12	78
15	3	82	15	6	79	15	9	79	15	12	73
20	3	77	20	6	74	20	9	71	20	12	68
25	3	72	25	6	69	25	9	66	25	12	63
30	3	67	30	6	64	30	9	61	30	12	58

Wt% is by weight percent of PVC.

### Property evaluation

#### Tensile properties and flexural properties

To assess the behaviour of the specimens when subjected to tensional stress, ultimate tensile strength, and elastic modulus were evaluated using the Universal testing machine (Instron 3369 Series) according to ASTM D3039M-17^48^ for procedure for tensile. Specimens were evaluated in commensuration with^48^ employing a load of 10 kg at room temperature (27 °C). Specimens with a gauge length of 150 mm as stipulated in D 638-14^49^ were used in estimating the tensile properties. Three specimens were evaluated and their average was presented as the result.

Flexural strength and modulus at the peak of hybrid composites developed was assessed by subjecting the specimens to a three-point bending load with the aid of a universal testing machine (Instron 3369 Series) in concert with ASTM D790-17^50^. Flexural properties were probed at room temperature while adopting a 0.3 mm/mm cross head speed and a constant strain rate of 10^–3^/s to fracture three specimens of 150 × 50 × 3 (mm^3^) dimension to obtain the average value for each weight fraction.

#### Relative density and water retention

Density of the composites developed was evaluated using the analytical weighing balance of high precision. Samples of each composites sample were measured to determine their mass and divided by its volume. Samples were immersed in a water medium of 250 cm^3^ for 7 days to evaluate this property. Water retention was appraised in accordance to ASTM D5229M-12^51^. Prior to the immersion, the initial mass of each composite sample was weighed using an analytical balance. The resulting weight gained was recorded for each day to estimate the total weight gained after 7 days.

#### Izod impact strength and compressive strength

Impact test was used to evaluate the toughness of the bamboo/coconut shell composite reinforced polyvinyl chloride composite. Three identical specimens were notched in a V-shape and subjected to an impact test in accordance with ASTM D256-10^52^ using an Izod impact testing machine. Samples were clamped and the pendulum was set at an angle of 165 ℃ to fracture the samples. In accordance to ASTM D 695-15^53^, the compressive strength was assessed by subjecting pure polyvinyl chloride and bamboo/coconut shell composite reinforced polyvinyl chloride composite to a compressive load. Three samples were assessed for each composition to determine their average value.

#### Hardness and wear loss index

Hardness of the pure polyvinyl chloride and polyvinylchloride based composites were appraised in congruent with ASTM D 785-08^54^ utilizing Rockwell hardness tester. Specimens of each weight fraction were positioned on the flat plate and indented using a diamond indenter to diminish the effect of surface. Specimens were indented 5 times on the surface to determine their average value which was employed for analysis.

Wear test was conducted to appraise the wear properties of the samples and for example, their sustainability in any application which involves contact and results in wear. This property was evaluated using Taber abraser (TSC-A016) in concert with ASTM D1044-13^55^. The equipment was operated at a speed of 150 rpm for a period of 10 min. Samples were weighed using an analytical balance prior to the test and the final weight was also recorded to estimate the wear undergone by each sample.

#### Thermal conductivity and electrical conductivity

Thermal conductivity was examined to determine the rate at which heat is transmitted from one side of the polymeric composite to the other. Lee’s disk apparatus was used for evaluating this property in accordance to ASTM E 1530^56^ by appraising samples with a diameter of 50 mm and 4 mm radius. While the efficiency of the samples to conduct electric charge was carried out in concert with ASTM D 257-14^57^ using Agilent 4339 B high resistance meter.

## Results and discussion

### Tensile properties

#### Tensile strength

**Figure 2:**
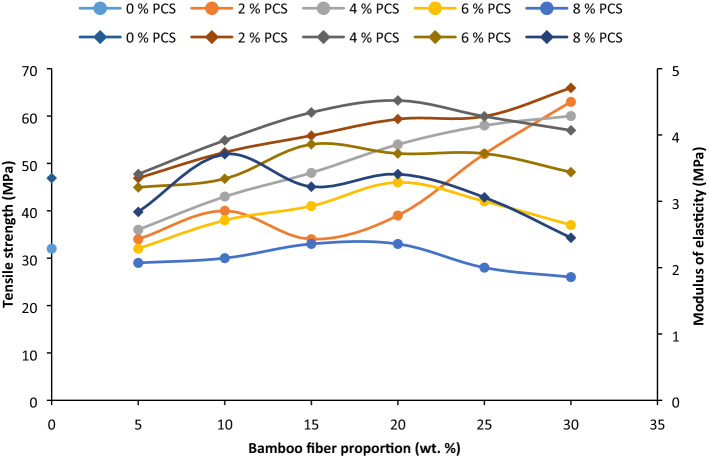
Influence of bamboo fiber fraction on tensile strength and modulus of elasticity for 0, 5, 10, 15, 20, 25 and 30 wt% fiber loading.

The behavior of the developed BF/PCS composite under tensile loading is as presented in Fig. 2. Variations in fiber and particulate loading affect the tensile behaviour of the composites. At 2% PCS, the tensile strength was observed to increase with fiber loading from 5 to 30 wt% presenting an increase of up to 63 MPa at 30 wt% BF when compared with 0/0 of BF/PCS. The rise may be due to coalesce of the fiber and filler particulate^58^. Good wettability of the particulate and BF fiber to the matrix enhanced adhesion, thereby inhibiting dislocation movement^59^. Incorporation of 4 wt% PCS, tensile strength appreciated in values from 5 to 20 wt% which can be linked to enhanced interfacial adhesion between fiber and matrix and proper filling of PCS. Admixture of 25–30 wt% BF and 4 wt% PCS resulted in a reduction in tensile strength observed occasioned by possible coagulation of particles (Fig. 8), of which the agglomeration point served as the region of storage of residual stress within the matrix^60^. Similarly, the same experience was noted when the particle portion was 6 and 8 wt%. General trend noted is that the tensile strength improved with fiber loading up to 20 wt% for particulate presence of 2–8 wt%, while it increased from 5 to 30 wt% for 2 wt% particle loading.

Increasing PCS proportion beyond 4 wt% amounted to lower tensile strength. Observation (Fig. 2) made with intermix of 2 wt% PCS and 0–30 wt% BF corroborates the observations made by^61–64^ in which tensile strength trended upward from 0 to 30 wt%. Also, the study of^65^ revealed an increase in tensile strength up to 6 wt% particulate cassava peel in the presence of 4.5 wt% palm kernel shell fiber.

#### Modulus of elasticity (MOE)

Modulus of elasticity as represented in Fig. 2 shows an appreciation in MOE values with increased fiber loading up to 30 wt% at 2 wt% of PCS addition on account of enhanced interfacial bonding and coalesce of fibers and particulate. Incorporation of 4, 6, and 8 wt% PCS, there was an uptrend in MOE fiber on integration of 5–20 wt% BF, while a reduction in MOE was observed on addition of 25–30 wt% BF, based on stress concentration and possible friction between particles and fiber, the consequence of which amounted to lower stiffness. Authors^65^ achieved higher MOE when 6 wt% particulate was incorporated in epoxy, the result of which affirms our finding of this study. In this case, integration of BF up to 20 wt% gave the maximum value for all particulate additions. Presence of fibers and particulates forms an obstacle to the free movement of dislocation^66^ effectuating the enhanced stiffness. According to^67^, the enhanced MOE as 10 wt% Doum Palm Shell Particle (sieved to 150 and 300 µm) in polypropylene. The result also confirms to the observation of^68^ in which egg shell powder improved the modulus of elasticity of polypropylene. MOE was noted to depreciate from 20 to 35 wt% particulate in^69^.

### Flexural properties

#### Flexural strength

**Figure 3:**
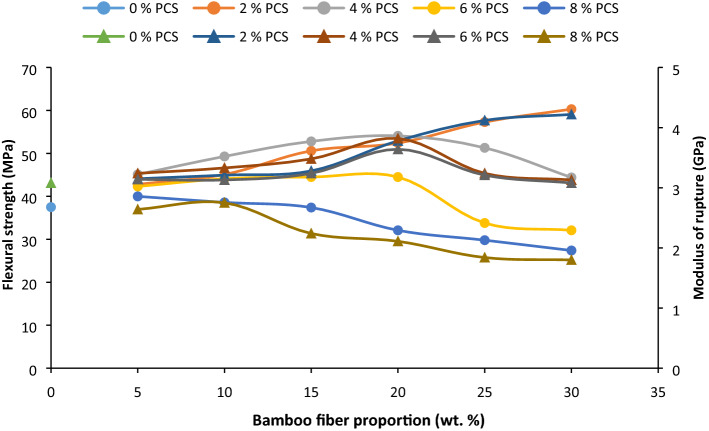
Influence of bamboo fiber fraction on flexural strength and modulus of rupture for 0, 5, 10, 15, 20, 25 and 30 wt% fiber loading.

From the plot in Fig. 3, it was noted that the flexural strength increased with fiber loading amounting to the attenuation of flexural strength on the addition of 2 wt% BF. This occurred by dint of coalesce between BF and PCS. Flexural strength on incorporation of 4, 6 wt% PCS amounted to accretion in FS value at fiber loading 5–20 wt% after which there was a decline in value (from 25 to 30 wt%). Inclusion of 8 wt% PCS impart a rise in flexural strength up to 10 wt% BF after which there was a progressive reduction in strength. The reason for this is on account of the agglomeration of filler particles (PCS), hence serving as a point of stress concentration. Authors^70^ assigned this event to poor stress transfer within interfaces. The highest flexural strength was 60 MPa at BF/PCS fraction of 30/3 wt%; a rise of 61% relative to proportion of 0/0 wt% additive. Highest value for flexural strength on addition of 4, 6, and 8 wt% PCS are 52.8, 44.5, and 38.6 MPa. This discloses a reduction in flexural strength with higher PCS proportion based on particulate agglomeration and fiber entanglement.

#### Modulus of rupture

Modulus of rupture (MOR) similarly followed the same pattern as unfolded in Fig. 3. MOR improved on the integration of 2 wt% PCS and fiber proportion 5–30 wt%. Enhancement in rigidity is attributable to enhanced interfacial bonding between fiber/particulate and matrix. Blending of 6 wt% of matrix showed enhancement in rigidity from 5 to 20 wt% BF; result which corroborates the observations made in^71,72^. Similar experience occurred when 6 wt% PCS in FM rose from 5 to 20 wt%, although at reducing value when compared with the value obtained under 4 wt%. Studies of^73–75^ affirm the result obtained. Utilization of bamboo fiber in^76^ presented an uptrend in modulus of rupture of epoxy-bamboo fiber composites up to 30 wt% BF affirming the usefulness of bamboo fiber in improving flexural rigidity. Reduction in rupture modulus from 25 to 30 wt% BF (2, 4, and 6 wt% PCS) and 15–30 wt% BF (12 wt% PCS) is linked to entanglement with the matrix^58^. The highest value was recorded on the blending of 30 wt% BF/3 wt% PCS value of 4.71 (0/0 wt% additive) by 53%.

### Density and water retention capacity

#### Relative density

**Figure 4:**
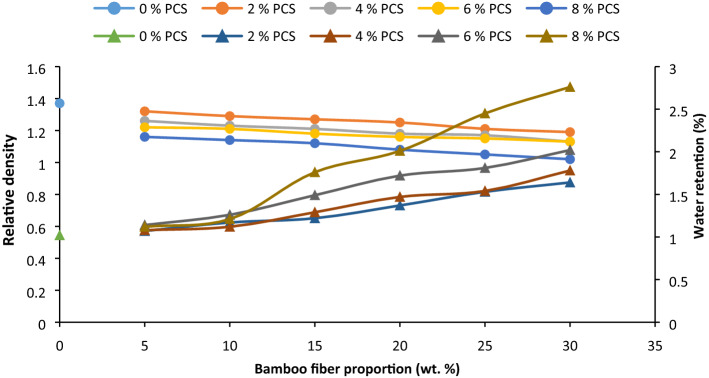
Influence of bamboo fiber fraction on relative density and water retention for 0, 5, 10, 15, 20, 25 and 30 wt% fiber loading.

The density of BF/BCF–PVC composite varied with additive proportion (Fig. 4). Average density of the sample containing 0/0 additive is 1.37, meanwhile this value reduced on the inclusion of 2, 4, 6, and 8 wt% PCS and 5 wt% fiber. It was observed that with increasing proportion of PCS, the density reduced owing to the light weight of particulate coconut shell. The results by Ref.^77^ show a lowering of density as the coconut shell powder filler increased, further corroborating in this study. In this study, the density of the composite depreciated with increasing fiber loading from 10 to 30 wt% BF. Incorporation of bamboo fiber and coconut shell powder resulted in lowering of densities. Lowering of density of BP/PCS PVC composite is beneficial in that the laptop must be light weight for easier carriage and portability. Diminishing values in density can be associated with lower density of fiber compared to the polymer.

#### Water retention

Water absorption results for samples reinforced with BF/PCS of varied proportion are as illustrated in Fig. 4. Water retention (%) trended upward with PCS proportion owing to the hydrophilic nature of the particulate^78^. Authors^79^ confirmed this result as par coconut shell powder addition. Water retention rose as PCS increased on the introduction of PCS from 2 to 6 wt% intermixed with BF from 5 to 30 wt%. A distinct finding made was that the incorporation of 8 wt% gave a steady increase when blended with 5 and 10% BF. Further blending of 15–20 wt% BF, there was an exponential rise in water retention accruing to the fact that PCS and BF fiber, which are hydrophilic, are occupying more volume resulting in higher water retention. Moreover, at that proportion, water penetration weakens the bond between fiber and matrix causing fiber detachment, hence leading to more water suction. Author^80^, studied the effect of coconut shell powder on the properties of polyurethane and he observed an increase in water absorption of the matrix with further addition of the biofiller. Just as obtained in^81,82^, water retention increased with BF addition. Further corroboration to this work is expressed in^83–85^.

### Impact strength and compressive strength

#### Impact strength

**Figure 5:**
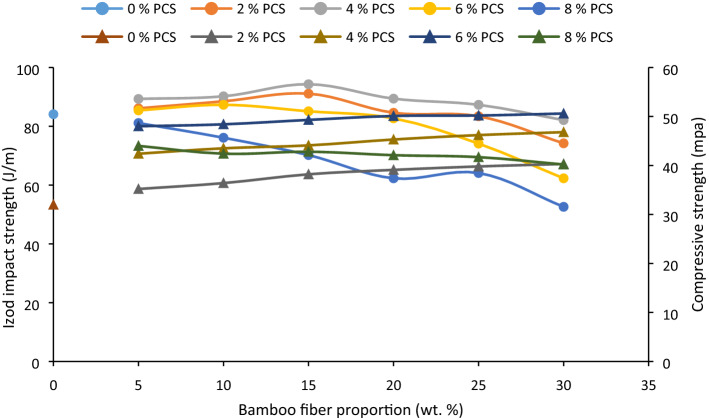
Influence of bamboo fiber fraction on Izod impact strength and compressive strength for 0, 5, 10, 15, 20, 25 and 30 wt% fiber loading.

Impact strength of the composites developed with respect to bamboo fiber/particulate coconut shell additive as presented in Fig. 5 was observed to rise with fiber loading from 5 to 30 wt% for samples knitted with 2 wt% PCS. Intermix of 5–15 wt% BF and 4 wt% PCS also enhanced the impact strength. Enhanced interfacial adhesion and even distribution of fillers within a matrix reducing interparticle distance provoke even stress distribution within the matrix thereby effectuating higher impact strength^86^. However, intermix of 20–30 wt% fiber and 2/4/6 wt% PCS resulted in depreciation in impact strength and this is ascribed to fiber agglomeration which serves as portion of stress concentration; thereby instigating brittleness within matrix. Interface at 6/8 wt% PCS at fiber loading of 5 and 10 wt% improved the impact strength and this can be credited to even stress distribution and enhanced interaction between fibers and particles under stress. However, at 6 and 8 wt% the the tendency for particle agglomeration increased, a consequence of which resulted in the lowering of strength at fiber loading 15–30 wt%. Observations made in this study can be linked to the study of^87^ in which the impact strength reduced with fiber loading up to 30 wt%. Similarly, wood fiber incorporated into polypropylene was reported to reduce the impact strength at increased fiber loading up to 40 wt%^88^.

The test was carried out on cylindrical samples of composites 40 mm in diameter and 80 mm in length and carried out as per ASTM D 695^53^. From Fig. 5, the compressive strength appreciated significantly with PCS loading from 2 to 8 wt% for all proportion of fiber content. Further observation is the marginal rise in compressive strength with fiber loading when considering the effect of the fiber on the strength under 2, 4, and 8% PCS. It can be inferred that particulate has a significant effect on the composites while BF has marginal effect on the compressive strength of the composite. Compressive strength was observed to peak at 55.2 MPa, an increase of 72.5% rise (relative to compressive strength of control 0 wt% PCS/BF) associated with the even distribution of PCS particles within the matrix. Compressive strength was detected to reduce at 8 wt% PCS loading. Agglomeration of particles is responsible for this, hence, during loading, residual stress were stored, amounting to lower strength against compressive stress. Observation depicted in^89^ corroborates the findings noted in this study as compressive strength reduced at 8 wt% fiber loading.

### Hardness and wear

#### Hardness

**Figure 6:**
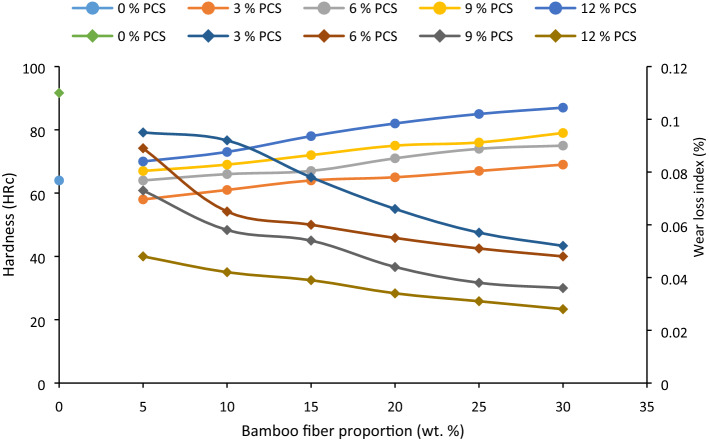
Influence of bamboo fiber fraction on hardness and wear loss index for 0, 5, 10, 15, 20, 25 and 30 wt% fiber loading.

Hardness was observed to increase with particulate and fiber bonding (Fig. 6). Enhanced interfacial adhesion promotes hardness which may be due to the strong adhesion of alkaline treated BF to PVC matrix. Additionally, PCS presence serves as a filler reducing the interparticle distance, repercussion of which amounted to improved hardness. Maximum hardness was attained at intermix at 30 wt% BF and 8 wt% PCS, a rise of 61%. Results obtained by Ref.^90^ revealed a progressive rise in hardness from 0 to 20% fiber used even as confirmed in this study.

The hardness depicted in this study conforms to the findings of^91^ where shore hardness was reported to increase with rising glass fiber/titania particles intermix. Authors^78^ also affirmed the increased Rockwell hardness of polymer matrix reinforced with coconut shell powder.

#### Wear

Wear resistance of the composite was evaluated by measuring weight loss during test. Lower weight loss depicts higher abrasion resistance (Fig. 6). Similar to the study of^92^ who studied the wear behaviour of polyvinyl pyrrolidone composite incorporated with date palm leave fiber. It was observed in the study that weight loss reduced with fiber loading irrespective of the load applied during test. Similar result was reported by Ref.^93^ the wear rate reduced as the percentage carbonized bone increased.

Increase in abrasion resistance with fiber and particulate loading is traceable to the enhanced cohesion within particles of the composites enabled by the fusion of coconut shell particles in the matrix. Fiber inclusions may also promotes abrasion resistance due to strong attachment to the matrix. The study of^94^ depicted a reduction in wear rate by increasing coir powder and coir fiber content. Wear rate was noted to decrease with increasing coir powder loading down to 25%. As observed in this study, wear loss was more pronounced with increasing powder presence than fiber, which is associated with ease of disengagement of particles than fiber^95^.

### Thermal and electrical conductivity

#### Thermal conductivity

**Figure 7:**
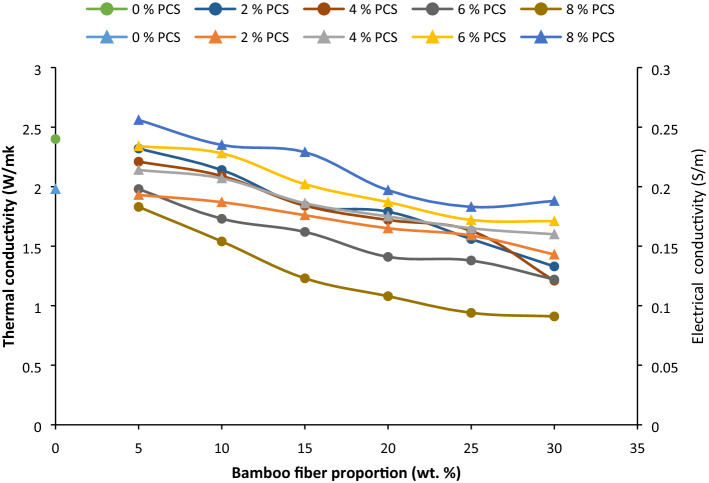
Influence of bamboo fiber fraction on thermal conductivity and electrical conductivity for 0, 5, 10, 15, 20, 25 and 30 wt% fiber loading.

Thermal conductivity of the composite developed increased marginally with PCS loading as presented in Fig. 7. Introduction of PCS led to a reduction of porosity promoting cohesion within particles in the matrix; thereby enhancing interparticle interaction. Thermal activation of particles amounts to excitation and gyration enabling the transfer of thermal energy from one particle to the next. Previous studies on the composite revealed an appreciation in thermal conductivity with a rise in copper particulate fraction^96^ and further confirmed by Ref.^97^.

Proportional rise in BF incorporated shows a lowering of thermal conductivity despite it has been treated. Natural fibers are characterized with inherent pores and higher volume presence in matrix, introduce a slight rise in porosity, and in effect, leads to a decrease in thermal conductivity owing to the distance between particles and the possible bridges in thermal transmission. This trend was in line with study carried out by Ref.^98^. Previous studies of^99^, revealed the decrease in thermal conductivity with increasing abaca fiber due to increase void with fiber loading, an observervation further corroborated in^100^.

#### Electrical conductivity

Coconut shell powder and bamboo fiber have poor electrical conductivity^101,102^. Presence of PCS in increasing proportion reduced the electrical conductivity (Fig. 7). With higher fiber fraction, the electrical conductivity also depreciated^103^. Lower electrical conductivity shows enhanced insulation properties, hence qualifying for insulation application. Lowest conductivity was reported at 30 wt% BF and 8 wt% PCS (0.91 S/m) gives 62% enhancement reduction in thermal conductivity with respect to control. From the report, increasing the proportion of BF and PCS enhances the insulation properties.

### Morphological analysis

**Figure 8:**
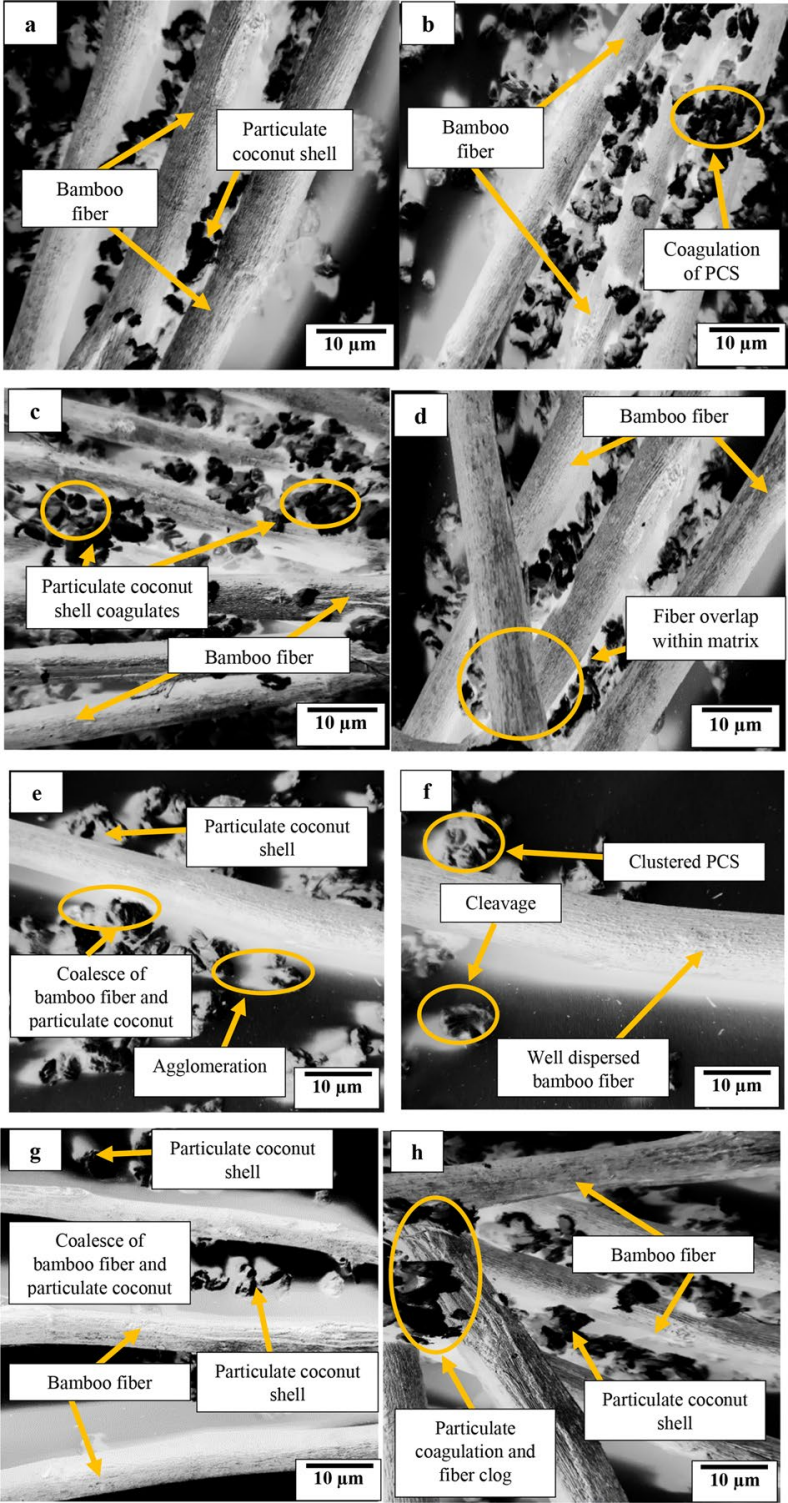
Morphological SEM image of compsoites amples reinforced with (**a**) 4 wt% PCS/20 wt% BF (**b**) 4 wt% PCS/25 wt% BF (**c**) 4 wt% PCS/30 wt% BF (**d**) 2 wt% PCS/20 wt% BF (**e**) 4 wt% PCS 3 wt% BF (**f**) 2 wt% PCS/5 wt% BF (**g**) 2 wt% PCS/30 wt% BF (**h**) 4 wt% PCS/30 wt% BF.

The representative morphological features of composite samples developed are as displayed in Fig. 8.

Figure 8 presents morphological images of selected samples representing the selected mixes of images presenting microstructural features. Particulate distribution in high quantity amounts to agglomeration of particles as indicated in Fig. 8b,c, and h. These points of agglomeration serve as the stress concentration points eventually amounting to the lowering of strength as observed under compressive strength and impact strength. Figure 8d reflected the fiber observed fiber overlap within the matrix, which eventually amounts to fiber clog as seen in Fig. 8h, the consequence of which reduces strength on the dint of uneven stress distribution. Coalesce of fibers and particulates (Fig. 8a,e,f, and g) indicates even stress distribution among particulates, fibers and matrix enhancing strength. Consequence of this was reflected in the increase in tensile and flexural strength, moduli of elasticity and rupture, impact strength, and compressive strength. The closeness of these particles by dint of reduced interparticle distances allows the transfer of heat when thermally agitated, eventually causing a rise in thermal conductivity as reflected in the uptrend in thermal conductivity with increasing PCS loading. However, based on the lower conductivity of the fiber, the conductivity reduced with increasing fiber loading. Inverse position was taken as the par electrical conductivity in that increasing proportion of fibers and particulates presented depreciation in the property value based on incoherence distribution of fibers and particulates as observed in the micrographs (Fig. 8c,d, and h).

## Conclusions

Treated bamboo fiber/particulate coconut shell hybrid PVC composite was examined for tensile, flexural, impact, and compressive strengths; moduli of elasticity and rupture. Other properties include hardness, wear loss index, water retention, thermal, and electrical conductivity. Results presented indicated that;

i. incorporation of 5, 10, 15, 20, 25, and 30 wt% bamboo fiber at 2 wt% constant particulate coconut shell resulted in enhancement of yield strength, modulus of elasticity, flexural strength and modulus of rupture, of which bamboo fiber proportion of 35 and 40 wt% resulted in strength depreciation. Similarly, particulate coconut shell addition of 4, 6, and 8 wt% instigates sdecrease in strength.
ii. thermal conductivity reduced consistently with increased fiber loading but slightly increase with increased particulate loading. Electrical conductivity reduced with increased fiber and particulate loading.
iii. Interfuse of 5, 10, 15 wt% fiber and 2 and 4 wt% particulate is effective in enhancing impact strength of which proportions beyond this is detrimental to impact strength of the composites. Compressive strength was better boasted with increasing fiber fraction and PCS amount but 8 wt% PCS amounted to depreciation in trend.
iv. blending of the two variable inputs; 5, 10, 15, 20, 25, and 30 wt% BF and 2 wt% PCS presented better enhancement in properties of composite developed and can be utilized in development of insulating pipes and ceiling boards.

## Data Availability

All data generated or analysed during this study are included in this published article.

## Code availability

No code was used for the computation of the data reported in this study.
